# Feasibility Study of Pelvic Helical IMRT for Elderly Patients with Endometrial Cancer

**DOI:** 10.1371/journal.pone.0113279

**Published:** 2014-11-25

**Authors:** Jean-Emmanuel Bibault, Philippe Nickers, Emmanuelle Tresch, Abel Cordoba, Eric Leblanc, Pauline Comte, Thomas Lacornerie, Eric Lartigau

**Affiliations:** 1 Academic Radiation Oncology Department, Oscar Lambret Comprehensive Cancer Center, 3 rue Frédéric Combemale, Lille, France; 2 Biostatistics Department, Oscar Lambret Comprehensive Cancer Center, 3 rue Frédéric Combemale, Lille, France; 3 Gynaecological Oncology Department, Oscar Lambret Comprehensive Cancer Center, 3 rue Frédéric Combemale, Lille, France; 4 Faculty of Medicine, University Lille 2, Lille, France; 5 ONCOLille, maison régionale de la recherche Clinique, Lille, France; Taipei Medical University, Taiwan

## Abstract

**Purpose:**

Standard treatment for early-stage endometrial cancer involves surgery (when possible) followed by brachytherapy or external-beam radiotherapy (EBRT) for high-risk tumors. EBRT is not without toxicity, meaning that it could be difficult to complete for elderly patients, who typically have decreased reserve and resistance to stressors.

**Patients and methods:**

Patients aged 70 and over treated between April 2009 and May 2013 for endometrial cancer and received IMRT (Intensity-Modulated Radiation Therapy) were included in this observational study. IMRT could be performed as adjuvant treatment or as an exclusive treatment for patients not amenable to surgery. The primary endpoints of this study were to assess the feasibility and toxicity of pelvic IMRT in this population. Secondary endpoints were to assess disease-specific survival, overall survival, and local control. Predictors of toxicity were also explored.

**Results:**

Forty seven consecutive patients were included in the analysis. Median age at diagnosis was 75 years (range, 70–89 years). Eleven patients were aged 80 years and older. Toxicities were found in thirty four patients (72%) during treatment. Among these, toxicity did not exceed grade 2 for 32 patients (68%). Two patients had a grade 3 toxicity (4%). Overall survival rates were 87% and 83% at 1 and 2 years, respectively. Six patients (12.8%) had a local relapse and nine others (19.1%) had distant relapse.

**Conclusions:**

Pelvic helical IMRT for patients aged 70 and older is feasible with full standard radiation doses, showing that age greater than 70 should not be considered as a reason not to perform optimal treatment.

## Introduction

Patients with endometrial cancer have a 95% 5-year specific survival rate when the disease is still localized. In the United States, endometrial cancer is the eighth cause of cancer death among women, with 7,780 deaths in 2009. Old age [1], ethnicity [2], comorbidities [3], and anemia [4] are the main risk factors of poor prognosis.

In a study published by Frick et al in 1973, old age was associated with shorter survival for patients with stage I disease who received the same treatment as others [5]. Aalders et al [6] also reported that the death rate was doubled for patients aged 60 years and over. More recently, Creutzberg et al showed that age over 60 years was associated with a three-fold increased risk of local relapse (p = 0.003) and higher disease-specific mortality (p = 0.02) [7]. The poor prognosis associated with old age is found across all tumor types [8] and could be explained by the lack of guidelines for elderly patients due to the underrepresentation of this population in clinical trials [9]. However, as life expectancy rises and as the chance of developing endometrial cancer increases with age, oncologists are required to find new ways to manage elderly patients in order to close the survival gap seen between middle-aged and elderly patients.

Standard treatment for early-stage endometrial cancer includes surgery (when possible) followed by brachytherapy or external-beam radiotherapy (EBRT) for high-risk tumors [10], [11]. Studies have shown that EBRT was not without toxicities [12], meaning that this treatment could be difficult to complete for elderly patients, who typically have decreased reserve and resistance to stressors. In the ASTEC/EN.5 study, the most significant toxicities were gastrointestinal or urogenital. They could potentially prevent the completion of the treatment or even, in the worst case, be life-threatening for medically fragile patients.

Intensity-modulated radiation therapy (IMRT) is still being evaluated for gynecological tumors [13]–[16]. No recommendation currently exists to choose IMRT in this setting. However, this high-precision technique could be used in order to treat frail, elderly patients who otherwise could not receive optimal treatments. This study was performed to evaluate the feasibility and toxicity of IMRT in elderly patients aged 70 years or older and treated for endometrial cancer.

## Patients and Methods

### Patients

Patients treated between April 2009 and May 2013 for endometrial cancer and received IMRT were included in this observational study approved by our internal review board. IMRT could be performed as an adjuvant or the exclusive treatment for patients not amenable to surgery. Patients could be included if they were aged 70 years or more with a Performance Status (PS) ≥2. Patients with a history of any other neoplastic disease within past 5 years, with chronic diarrhea, with a history of inflammatory bowel disease, or peritonitis were excluded.

### Follow-up

Toxicity was assessed using the NCI-CTCAE (National Cancer Institute Common Terminology Criteria for Adverse Events) v4.0 scale. Secondary objective was to assess disease-specific survival, overall survival and local control. The RECIST (Response Evaluation Criteria In Solid Tumors) criteria were used to assess treatment efficacy [17].

### Treatment planning

CT-Scan were contoured according to the RTOG (Radiation Therapy Oncology Group) guidelines [18]. PTV (Planning Target Volume) was obtained after an automatic expansion of 5 mm on the CTV (Clinical Target Volume). At least 95% of the PTV received 95% of the prescribed dose. Patients treated with adjuvant IMRT for type I N0 tumors received 45 Gy. Patients with type II tumors or lymph node metastases received 50.5 Gy. For patients not amenable to surgery and treated exclusively with IMRT, GTV (Gross Tumor Volume) included the uterus and macroscopic lymph nodes. CTV included GTV and the upper one third of the vagina. A 5-mm margin was applied to the CTV to obtain the PTV. Sixty Gy to the CTV with a boost of 10 Gy to the GTV were prescribed for these patients. The rectum, sigmoid colon, small bowel cavity, bladder, and skin were contoured as organs at risk. Dose constraints for the organs at risk are presented in table S1 in file S1.

### Treatment delivery

Treatments were performed on two identical Tomotherapy systems (Accuray Incorporated, Sunnyvale, CA) with daily MVCT (Megavoltage Computed Tomography) to verify the positioning of the patients and organs at risk. Patients were asked to have a digestive and urinary preparation (empty rectum and half-full bladder). Laxatives and strict instructions were given to the patients in order to achieve a good preparation for treatment before the planning CT-Scan and before every treatment session.

### Statistical analysis

Dose Volume Histograms (DVHs) for PTV, rectum, sigmoid colon, small bowel, and bladder were exported from the Tomotherapy planning station to perform correlations with the reported toxicity. Survival was calculated from the date the treatment was finished to the date of death or relapse. Predictive factors of toxicities were explored using the Wilcoxon Mann-Whitney test. Factors tested include previous surgery, treatment volume (PTV), D2%, D50%, D2cc, D10cc, D30cc to small bowel, sigmoid colon and rectum. Overall survival, disease-specific survival, and local control were calculated using the Kaplan-Meier method. Correlations between survival and patient characteristics were calculated using the logrank test with p value<0.05. All analyses were performed with Stata v11.2 (StataCorp. 2009. Stata Statistical Software: Release 11. College Station, TX: StataCorp LP.)

### Ethics

This study was approved by the internal ethic board of our institution (Clinical Trial Commission; “Commission interne des études cliniques”). Our institutional review board waived the need for written informed consent from the participants. French laws (Data, data-collection and freedom law, January, 6th 1978) state that in case of single-centre, retrospective study based on already recorded and stored data, there is no need of specific written informed consent.

All patients have been orally informed about the potential use of their collected data for future research. Agreement N1034071 was obtained from the “National Commission about Data-collection and Freedom” (“Commission Nationale Informatique et Liberte'”) for the conduct of this study.

## Results

### Patients and treatments

Forty-seven consecutive patients aged 70 and older were treated with IMRT between April 2009 and May 2013 at Oscar Lambret Cancer Center. Median age at diagnosis was 75 years (range, 70–89). Thirty six patients were between 70 and 80 years old (76.6%). Eleven patients were 80 or older. Forty-four patients were treated for type I endometrial cancer and nine for a type II tumor. Thirty-seven patients (86.1%) had a WHO score ≤1. Patients' characteristics are presented in table 1. Forty-one patients received adjuvant, and three patients IMRT monotherapy because they were not candidates for surgery. One patient undergoing adjuvant external radiotherapy did not complete the treatment due to a grade 3 digestive toxicity. Treatment was stopped at 38.64 Gy and could not be finished. Ten patients received concurrent chemotherapy (cisplatin 50 mg/m2 i.v., 2 cycles during radiotherapy, 3-week interval). Patients received high-dose-rate brachytherapy to the vaginal cuff after IMRT was completed (two fractions of 6 Gy prescribed to the vaginal mucosa).

**Table 1:** Patients characteristics.

Patient characteristics (N = 47)	n	%
**Age**		
<80 years old	36	76.6%
≥80 years old	11	23.4%
**Histology**		
Type I	38	80.9%
Type II	9	19.1%
**FIGO**		
1a	7	14.9%
1b	12	25.5%
1c	3	6.4%
2a	2	4.3%
2b	6	12.8%
3a	4	8.5%
3b	3	6.4%
3c	8	17.0%
4a	1	2.1%
4b	1	2.1%
**Grade (N = 42)**		
1	14	33.3%
2	15	35.7%
3	13	31.0%
**BMI (kg/m^2^) (N = 27)**		
<25	6	22.2%
25–30: overweight	9	33.3%
≥30: obesity	12	44.4%
**WHO (N = 43)**		
0	7	16.3%
1	30	69.8%
2	6	13.9%

Treatment duration, total dose, number of fractions, and dose/fraction are shown in table 2. Median D98% for PTV was 43.6 Gy. Complete dosimetric data is shown in table S3 in file S1.

**Table 2:** Treatment characteristics.

	Median	Min	-	Max	Mean	Standard deviation
**Time from diagnosis to IMRT (days or months)**	2.9 m	11days	-	46 m	4.8 m	8.3 m
**Duration of IMRT (days)**	40	33	-	49	40	4
**Total dose (Gy)**	45.0	38.6	-	68.9	48.6	6.3
**Number of fractions**	25	23	-	33	26	2
**Dose per fraction (Gy)**	1.8	1.7	-	2.3	1.8	0.1

### Acute toxicity

Toxicities were found in 34 patients (72%) during treatment. Among these, toxicity did not exceed grade 2 for 11 patients (23%). Two patients had a grade 3 toxicity (4%). The most frequent toxicities were: digestive (51%), urologic (21%), fatigue (15%), and pain (13%). A Grade 3 adverse event was encountered in a patient receiving chemotherapy who had anemia requiring blood transfusion and in another patient undergoing adjuvant external radiotherapy who had a grade 3 diarrhea that led to treatment interruption. Toxicities are reported in table 3.

**Table 3:** Toxicities of pelvic helical IMRT in patients aged 70 years and older.

Toxicities	n	%
**Toxicities (all types)**	34	72,3%
Maximum grade by patient:		
Grade 1	21	44,7%
Grade 2	11	23,3%
Grade 3	2	4,3%
**Digestive toxicities**	24	51,1%
Grade 1	13	27,7%
Grade 2	10	21,3%
Grade 3	1	2,1%
**Urologic toxicities**	10	21,3%
Grade 1	9	19,1%
Grade 2	1	2,1%
**Fatigue**: Grade 1	7	14,9%
**Pain**: Grade 1	6	12,8%
**Nausea**: Grade 1	4	8,5%
**Metrorrhagia**: Grade 1	2	4,3%
**Dermitis**: Grade 1	1	2,1%
**Hematologic**: Grade 3	1	2,1%

**Figure 1:**
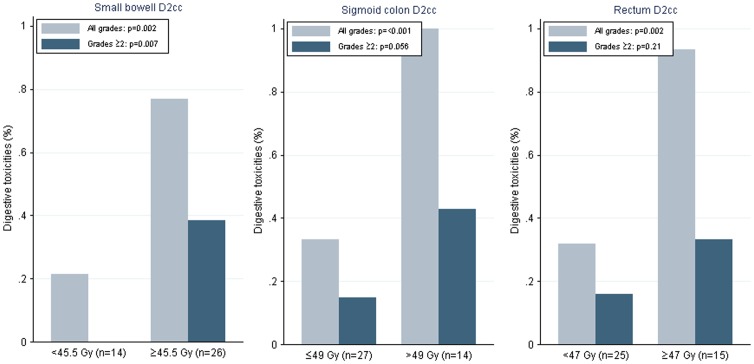
Dose thresholds for the small bowel, sigmoid colon, and rectum for digestive toxicities in pelvic tomotherapy for elderly patients.

Dosimetric data were available for 41 patients. Median cumulative doses when these toxicities appeared are shown in table S2 in file S1. Median cumulative dose when toxicities appeared were 27.9 Gy for digestive toxicities, 44 Gy for urologic toxicities, and 36 Gy for cases of pelvic pain. There was no statistical association between previous surgery and toxicity (p = 1). A statistically significant correlation was found between size of the PTV and urinary toxicity (p = 0.021). Regarding digestive toxicity, maximum spot dose to the small bowel, sigmoid, and rectum were found liable. Dosimetric thresholds were calculated for each organ at risk for all digestive toxicities and grade 2 and higher toxicities. Results are shown in figure 1. For small bowel, a maximum dose of 45.5 Gy to 2 cc was linked to a higher number of grade 2 digestive toxicities (p = 0.007). The maximum dose of 49 Gy to 2 cc of sigmoid colon was linked to a higher rate of toxicities (all grade, p<0.001). For the rectum, a maximum dose of 47 Gy to 2cc of the volume was linked to higher rates of toxicities (all grades, p = 0.002).

### Survival

**Figure 2:**
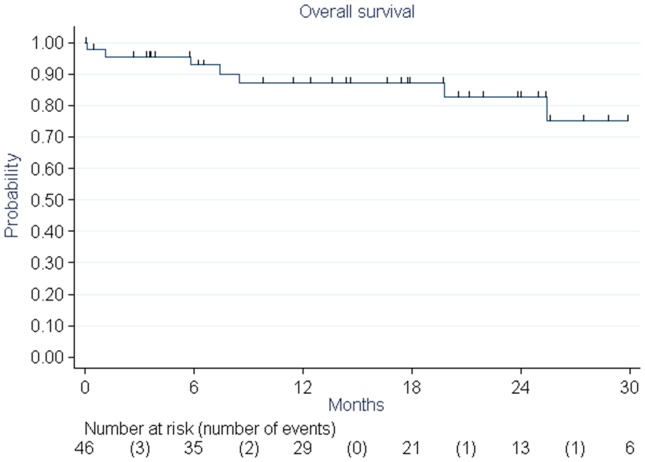
Overall survival of elderly patients treated with pelvic helical IMRT for endometrial cancer estimated with the Kaplan-Meier method.

**Figure 3:**
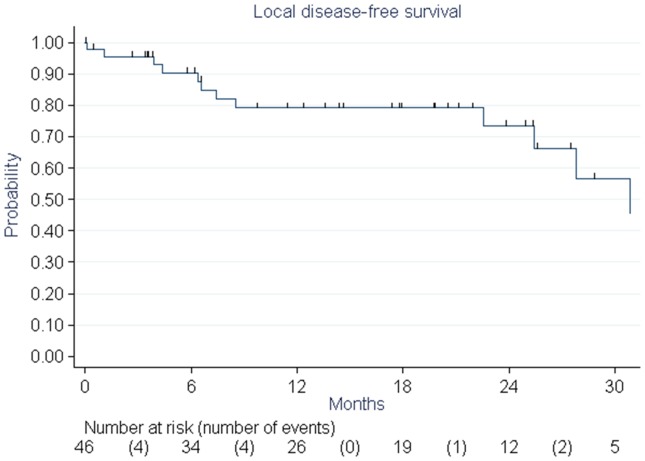
Local control of elderly patients treated with pelvic helical IMRT for endometrial cancer estimated with the Kaplan-Meier method.

Nine patients (19%) died during follow-up. Five (11%) of them died of cancer progression. Overall survival rates were 87% and 83% at 1 and 2 years, respectively (figure 2). Disease-free survival rates were 67% and 54% at 1 and 2 years, respectively. Six patients (12.8%) had a local relapse (figure 3) and nine others (19.1%) had distant metastases. Age, obesity, WHO (World Health Organization) status, FIGO (International Federation of Gynecology and Obstetrics), grade, dose received by the PTV, and concomitant chemotherapy were tested as prognostic factors and no statistically significant relationship was found.

## Discussion

Since 1973, several studies have described older age as a poor prognosis factor for patients with endometrial cancer [5]–[7]. However, the true influence of age on overall survival is still subject to debate. Two recent studies showed that advanced age was a determinant of poor prognosis in these patients [19]–[21]. But in 2011, Fleming et al. performed a retrospective analysis on 338 patients with stage IA to IIB endometrioid endometrial adenocarcinoma and showed that after adjusting for other poor prognosis factors such as grade and coronary artery disease, age was no longer a significant variable for overall survival (p = 0.17) [22]. The lower overall survival in this population could be due to comorbidities rather than endometrial cancer. Therefore chronological age should not change treatment indications, which should be tailored individually based on WHO Performance Status and comorbidities. In line with this, older age by itself should not be a contraindication for the proper surgical treatment of elderly women with endometrial cancer since perioperative complications rates are similar in younger patients [23]. A SEER (Surveillance, Epidemiology, and End Results Program) analysis performed between 1992 and 2002 determined that the poor prognosis associated with advanced age could be in part linked to the decreased frequency of surgical treatment in older patients [24].

Adjuvant radiation therapy is indicated in high-risk patients with endometrial cancer treated with surgery [12]. The Post Operative Radiation Therapy in Endometrial Cancer (PORTEC-1) trial [25] showed that 3D-CRT decreased the 15-year actuarial locoregional recurrence rates to 5.8% (vs 15.5%) (p<0.001) without significant difference on the 15-year overall survival rates (52% versus 60%, p = 0.14). Despite EBRT's efficacy in reducing locoregional recurrence, the authors concluded that EBRT should be avoided in patients wilh low and intermediate-risk endometrial cancer because of higher rates of RT-related complications such as urinary incontinence, diarrhea, and fecal leakage. The PORTEC-2 trial compared vaginal brachytherapy (VBT) with EBRT for postoperative adjuvant treatment and showed that VBT was effective in ensuring vaginal control with fewer gastrointestinal toxic effects [26]. The standard radiotherapy techniques used to treat the pelvis after hysterectomy for endometrial corpus carcinoma consisted in these trials of two- to four-field treatments. The entire content of the pelvis is irradiated to the prescribed dose, including the bladder, small bowel, sigmoid colon, and rectum. The compliance to treatment can be difficult to achieve with this technique, especially in older patients [27]. Alektiar et al published a study [19] to determine the influence of old age (>70 years) on outcome in a group of patients with endometrial carcinoma who were treated with simple hysterectomy followed by adjuvant 3D-CRT (3D Conformal Radiation Therapy). They reported a 20% rate of non-compliant 3D-CRT and showed that the recurrence rate was 7% in the patients with a treatment deviation compared with 3% in patients without a deviation. This result could suggest that part of the poor prognosis associated with old age in endometrial cancer could be explained by non-optimal treatment because radiation oncologists can be reluctant to perform full-dose treatment and decide to reduce the prescribed dose or even avoid radiation therapy altogether.

Tomotherapy and IMRT can deliver a high dose of radiation to an irregular concave-shaped clinical target volume, sparing the adjacent normal tissues [28]. IMRT has been increasingly used to treat gynecologic malignancies. Several studies have established that this technique can reduce the volume of organs at risk irradiated compared to 3DRT in the adjuvant setting where small bowel are often found in the pelvis [29]–[32].

While we did not gather the clinical and dosimetric data for our younger patients, we can still compare the toxicity with a study published by Barillot et al. [16] This phase II trial assessed the impact of post operative intensity modulated radiotherapy on acute gastro-intestinal toxicity for patients with endometrial cancer. From May 2008 to April 2010, 49 patients from 6 centres were included. 46 cases were available for analysis. Median age was 65.5 y.o.±8.9 years (ten years younger than in our study). Thirty six patients (75%) received an additional vaginal vault boost of 6–10 Gy delivered by HDR (High Dose Rate) brachytherapy in 1 or 2 fractions. 27% of the patients (95% CI 14.5–39.7%) developed at least 1 gastro-intestinal grade 2 adverse event (diarrhea in 92% of cases), which mainly occurred at week 4 and week 5 (which is consistent with our own data). No event corresponding to grade 3 or above was recorded. Five patients complained about late gastro-intestinal events (grade 1 diarrhea). Early toxicity also included cystitis or urinary frequency (92% of patients). Overall, in this population, toxicity was very low, like in the study presented here.

Another potential benefit of IMRT for adjuvant or exclusive treatment of endometrial cancer is the sparing of the pelvic bone marrow. RTOG 0418 is a phase 2 study testing the feasibility of delivering postoperative IMRT for cervical and endometrial cancer in which 43 patients with endometrial cancer were included [33]. At the end of the trial, the authors concluded that limiting the volume of irradiated bone marrow was associated with reduced rates of hematologic toxicities and could improve tolerance to chemotherapy, which is consistent with our results. Only one patient had grade 3 hemotoxicity requiring blood transfusion.

We believe the clinical advantages of IMRT should be employed for older patients for several reasons: to be able to prescribe the full radiation dose without deviation from the standard treatment; to better tailor treatment to spare digestive organs and bone marrow; and to prevent any exacerbation of pre-existing comorbidities in this population. Our study is the first to assess the feasibility of pelvic IMRT in older patients. All patients but one finished their treatment. A majority (n = 34, 72%) of our patients had toxicities: most of them were grade 1 (n = 21, 44.7%). Eleven patients (23%) had grade 2 toxicities and 2 patients had grade 3 hematologic (n = 1, 2%) and digestive toxicity (n = 1, 2%). Most frequent adverse events were digestive (n = 24, 51%) and urologic (n = 10, 21.3%). These rates cannot be compared to the toxicity reported in the PORTEC-1 trial because that trial did not use the CTCAE scale to report and grade these symptoms. PORTEC-2 [26] and the GOG phase III [34] trials on the other hand used the same scale but focused the analysis on long-term results and did not specifically report on acute toxicity, which is the major limit to pelvic radiation therapy in an older population.

The limitations of our study are the limited follow-up of the patients and the retrospective nature of the analysis but the toxicity rates are acceptable and the results seem to validate the dose constraints we used for the study (table S1 in file S1). It should be noted that this treatment was made possible with strict digestive and urinary preparation instructions and daily MV-CT repositioning and could prove difficult to reproduce in another setting. Our study also showed the importance of the maximum spot dose of the small bowel, sigmoid colon and rectum. These dosimetric factors should be carefully evaluated and considered for treatment planning in this population.

## Conclusions

Pelvic helical IMRT for patients aged 70 and older is feasible with full standard radiation doses, when strict dose constraints can be applied to pelvic digestive tissues and bladder. No major toxicities were reported. These dose constraints should be applied in order to minimize toxicities and allow full treatment completion. In this study, patients' survival was in line with survival rates reported in other studies, showing that age greater than 70 should not be considered as a reason not to perform optimal treatment. A phase III randomized multi-center trial (NCT01641497) is now underway to compare IMRT to 3D-CRT in this population and further validate these results.

## Supporting Information

File S1 Contains Tables S1–S3.
(DOC)
