# Supplementary material for: Feasibility Study of Pelvic Helical IMRT for Elderly Patients with Endometrial Cancer
Source: PLoS One. 2014 Nov 25;9(11):e113279. doi: 10.1371/journal.pone.0113279 (PMC4244154; doi:10.1371/journal.pone.0113279)
Supplement: File S1 — Contains Tables S1–S3. (DOC) [file pone.0113279.s001.doc]

**Supporting information**

| **Organs** | **Dose constraints** |
| --- | --- |
| Small bowel | 200 cc < 40 Gy  50 cc <45 Gy  Dmax < 50 Gy |
| Sigmoid colon / Rectum | 2 cc < 60 Gy  20% < 45 Gy  50% < 40 Gy |
| Bladder |
| Bone | Dmax < 45 Gy |
| Skin (6 mm internal expansion) | Dmax < 30 Gy  10 % < 50 Gy |

Table S1 - Dose constraints used for pelvic helical IMRT

| **Toxicities** | **N** | **Médiane** | **(Min** | **-** | **Max)** |
| --- | --- | --- | --- | --- | --- |
| Digestive | 20 | 27.9 | (14.0 | - | 50.4) |
| Urologic | 7 | 44.0 | (23.4 | - | 50.4) |
| Fatigue | 2 | 33.3 | (16.2 | - | 50.4) |
| Pelvic pain | 3 | 36.0 | (18.0 | - | 45.0) |
| Nausea | 1 | 50.4 |  |  |  |
| Metrorrhagia | 2 | 21.0 | (10.0 | - | 32.0) |

Table S2 - Cumulative dose when toxicities appeared

|  | **Median** | **(Min** | **-** | **Max)** | **Mean** | **(Standard Deviation)** |
| --- | --- | --- | --- | --- | --- | --- |
| **PTV** |  |  |  |  |  |  |
| Total volume (cc) | 613.95 | (66.03 | - | 1,759.07) | 620.38 | (386.72) |
| D98% (Gy) | 43.60 | (40.75 | - | 68.90) | 45.18 | (4.55) |
| **Bladder** |  |  |  |  |  |  |
| Total volume (cc) | 177.41 | (39.63 | - | 693.00) | 226.14 | (148.82) |
| D2% (Gy) | 45.25 | (41.06 | - | 68.50) | 47.73 | (4.85) |
| D50% (Gy) | 27.00 | (11.94 | - | 40.46) | 28.04 | (6.18) |
| D98% (Gy) | 17.12 | (2.03 | - | 29.41) | 17.31 | (5.55) |
| D2cc (Gy) | 45.50 | (41.50 | - | 69.30) | 48.13 | (4.99) |
| D10cc (Gy) | 44.51 | (31.95 | - | 66.91) | 46.05 | (5.70) |
| D30cc (Gy) | 42.33 | (22.04 | - | 60.55) | 41.00 | (8.09) |
| D50cc (Gy) | 38.30 | (15.50 | - | 53.99) | 36.62 | (8.96) |
| **Small bowel** |  |  |  |  |  |  |
| Total volume (cc) | 2,059.24 | (592.62 | - | 8,587.30) | 2,605.87 | (1,774.76) |
| D2% (Gy) | 43.37 | (21.81 | - | 53.74) | 43.10 | (5.02) |
| D50% (Gy) | 16.32 | (0.36 | - | 29.89) | 16.32 | (5.51) |
| D98% (Gy) | 4.05 | (0.18 | - | 15.21) | 5.12 | (4.42) |
| D2cc (Gy) | 46.02 | (42.19 | - | 68.89) | 48.20 | (4.81) |
| D10cc (Gy) | 45.19 | (30.93 | - | 61.98) | 46.56 | (4.81) |
| D30cc (Gy) | 43.85 | (17.52 | - | 56.72) | 44.01 | (5.82) |
| D50cc (Gy) | 42.48 | (12.15 | - | 54.66) | 42.22 | (6.51) |
| **Rectum** |  |  |  |  |  |  |
| Total volume (cc) | 67.49 | (17.03 | - | 222.36) | 78.03 | (49.81) |
| D2% (Gy) | 46.34 | (13.20 | - | 67.30) | 46.96 | (7.37) |
| D50% (Gy) | 30.63 | (3.59 | - | 49.36) | 29.86 | (7.86) |
| D98% (Gy) | 10.48 | (1.61 | - | 33.32) | 11.48 | (8.01) |
| D2cc (Gy) | 45.75 | (9.91 | - | 68.97) | 46.40 | (7.97) |
| D10cc (Gy) | 42.91 | (4.74 | - | 65.21) | 41.89 | (9.18) |
| D30cc (Gy) | 33.13 | (1.51 | - | 55.17) | 32.59 | (11.17) |
| D50cc (Gy) | 26.75 | (5.05 | - | 49.94) | 27.68 | (11.10) |
| **Sigmoid colon** |  |  |  |  |  |  |
| Total volume (cc) | 82.79 | (35.87 | - | 374.59) | 112.14 | (72.42) |
| D2% (Gy) | 45.07 | (40.62 | - | 64.28) | 46.84 | (4.64) |
| D50% (Gy) | 31.75 | (13.75 | - | 42.85) | 31.58 | (6.42) |
| D98% (Gy) | 18.50 | (0.50 | - | 32.08) | 18.98 | (7.07) |
| D2cc (Gy) | 45.53 | (39.65 | - | 64.06) | 46.69 | (4.68) |
| D10cc (Gy) | 44.00 | (32.50 | - | 58.20) | 43.30 | (5.17) |
| D30cc (Gy) | 38.27 | (21.75 | - | 51.44) | 36.23 | (7.03) |
| D50cc (Gy) | 32.50 | (12.57 | - | 46.14) | 31.42 | (7.88) |

Table S3 - Dosimetric data (cc = cubic centimeter)
